# Resistance to Artesunate + Mefloquine does not incurin fitness cost in malaria parasites

**DOI:** 10.1186/1475-2875-9-S2-P43

**Published:** 2010-10-20

**Authors:** Louise A Rodrigues, Gisela Henriques, Pedro Cravo

**Affiliations:** 1 Molecular Biology Unity, Institute of Hygene and Tropical Medicine, Universidade Nova de Lisboa, Portugal

## Background

Resistance to almost all available anti-malarials poses a large threat for the effective control of Malaria. The only remaining exceptions are the artemisinin (ART) derivatives. However, there is now evidence that appearance of resistance to ART either administered alone or in combination with other anti-malarials may become a reality.

Although resistance to treatment is an obstacle for controlling the disease, in many biological models increased drug resistance is often associated with a biological cost. Similarly, in malaria parasites, resistance to anti-malarial drugs such as chloroquine (CQ) [1] and mefloquine (MF) [2] has been reported as causing a reduction in parasite's fitness associated with lower asexual growth rates and/or reduced transmission efficiency.

By using an artesunate (ATN)-resistant parasite, AS-ATN, we have selected a parasite clone (AS-ATNMF-1) which is resistance to the artesunate + mefloquine (ATN + MF) version of Artemisinin Combination Therapy [3]. AS-ATNMF-1 is also resistant to each component of the combination when administered separately. AS-ATNMF-1 was obtained after twenty-seven consecutive sub-inoculations of AS-ATN in mice treated with the ATN + MF combination. Since consecutive sub-inoculations into mice have been shown to cause increased virulence in malaria parasites [4], AS-ATN was also sub-inoculated twenty-seven times into untreated mice in parallel. The resulting line was named AS-ATN27P.

In order to assess the fitness cost associated with resistance to the combination ATN + MF, AS-ATNMF-1 parasites were grown with the AS-ATN parental clone or with AS-ATN27P within the same host in absence of drug pressure, for 14 days.

The proportions of each parasite parasite within were determined along time by Proportional Sequencing [5].

## Results

When in competition within the same host, AS-ATNMF-1 outgrows the unpassaged progenitor AS-ATN. However the same is not observed when grown in competition with the passaged parasites AS-ATN27P.

## Conclusions

Our data suggest that consecutive passaging may have induced an improvement in AS-ATNMF-1 fitness and that resistance to the combination ATN + MF seem to have no negative impact on the growth of these parasites. These results may have implications regarding the maintenance of ACT-resistant parasites in the absence of drug pressure.
